# Assessing heart rate fragmentation to predict atrial fibrillation in the general population aged 65: the PROOF-AF study

**DOI:** 10.1093/ehjopen/oeaf030

**Published:** 2025-03-19

**Authors:** Jean-Baptiste Guichard, David Hupin, Vincent Pichot, Mathieu Berger, Sébastien Celle, Roger Borràs, Ivo Roca-Luque, Lluís Mont, Antoine Da Costa, Jean-Claude Barthélémy, Frédéric Roche

**Affiliations:** Institut Clínic Cardiovascular (ICCV), Hospital Clínic, Universitat de Barcelona, Carrer Villaroel, 170, 08036 Barcelona, Catalonia, Spain; Fundació de Recerca Clínic Barcelona - Institut d’Investigacions Biomèdiques August Pi i Sunyer (FRCB-IDIBAPS), Carrer Rosselló 149-153, 08036 Barcelona, Catalonia, Spain; Centro de Investigación Biomédica en Red de Enfermedades Cardiovasculares (CIBERCV), Instituto de Salud Carlos III, Av. Monforte de Lemos 3-5, 28029 Madrid, Spain; INSERM, SAINBIOSE U1059, Campus Santé Innovation, 10 rue de la Marandière, 42270 Saint-Priest-en-Jarez, France; Cardiology Department, University Hospital of Saint-Étienne, 42 Av. Albert Raymond, 42270 Saint-Priest-en-Jarez, France; INSERM, SAINBIOSE U1059, Campus Santé Innovation, 10 rue de la Marandière, 42270 Saint-Priest-en-Jarez, France; Department of Clinical and Exercise Physiology, University Hospital of Saint-Étienne, 42 Av. Albert Raymond, 42270 Saint-Priest-en-Jarez, France; INSERM, SAINBIOSE U1059, Campus Santé Innovation, 10 rue de la Marandière, 42270 Saint-Priest-en-Jarez, France; INSERM, SAINBIOSE U1059, Campus Santé Innovation, 10 rue de la Marandière, 42270 Saint-Priest-en-Jarez, France; Department of Clinical and Exercise Physiology, University Hospital of Saint-Étienne, 42 Av. Albert Raymond, 42270 Saint-Priest-en-Jarez, France; INSERM, SAINBIOSE U1059, Campus Santé Innovation, 10 rue de la Marandière, 42270 Saint-Priest-en-Jarez, France; Institut Clínic Cardiovascular (ICCV), Hospital Clínic, Universitat de Barcelona, Carrer Villaroel, 170, 08036 Barcelona, Catalonia, Spain; Centro de Investigación Biomédica en Red e Salud Mental (CIBERSAM), Instituto de Salud Carlos III, Av. Monforte de Lemos 3-5, 28029 Madrid, Spain; Institut Clínic Cardiovascular (ICCV), Hospital Clínic, Universitat de Barcelona, Carrer Villaroel, 170, 08036 Barcelona, Catalonia, Spain; Fundació de Recerca Clínic Barcelona - Institut d’Investigacions Biomèdiques August Pi i Sunyer (FRCB-IDIBAPS), Carrer Rosselló 149-153, 08036 Barcelona, Catalonia, Spain; Centro de Investigación Biomédica en Red de Enfermedades Cardiovasculares (CIBERCV), Instituto de Salud Carlos III, Av. Monforte de Lemos 3-5, 28029 Madrid, Spain; Institut Clínic Cardiovascular (ICCV), Hospital Clínic, Universitat de Barcelona, Carrer Villaroel, 170, 08036 Barcelona, Catalonia, Spain; Fundació de Recerca Clínic Barcelona - Institut d’Investigacions Biomèdiques August Pi i Sunyer (FRCB-IDIBAPS), Carrer Rosselló 149-153, 08036 Barcelona, Catalonia, Spain; Centro de Investigación Biomédica en Red de Enfermedades Cardiovasculares (CIBERCV), Instituto de Salud Carlos III, Av. Monforte de Lemos 3-5, 28029 Madrid, Spain; INSERM, SAINBIOSE U1059, Campus Santé Innovation, 10 rue de la Marandière, 42270 Saint-Priest-en-Jarez, France; Cardiology Department, University Hospital of Saint-Étienne, 42 Av. Albert Raymond, 42270 Saint-Priest-en-Jarez, France; INSERM, SAINBIOSE U1059, Campus Santé Innovation, 10 rue de la Marandière, 42270 Saint-Priest-en-Jarez, France; Department of Clinical and Exercise Physiology, University Hospital of Saint-Étienne, 42 Av. Albert Raymond, 42270 Saint-Priest-en-Jarez, France; INSERM, SAINBIOSE U1059, Campus Santé Innovation, 10 rue de la Marandière, 42270 Saint-Priest-en-Jarez, France; Department of Clinical and Exercise Physiology, University Hospital of Saint-Étienne, 42 Av. Albert Raymond, 42270 Saint-Priest-en-Jarez, France

**Keywords:** Atrial fibrillation, Autonomic nervous system, Heart rate variability, Atrial cardiomyopathy, Heart rate fragmentation

## Abstract

**Aims:**

Screening the general population aged 65 for atrial fibrillation (AF) has been proposed as a preventive measure against potential complications. Metrics derived from heart rate variability (HRV) that depict heart rate fragmentation (HRF) have been suggested to reflect autonomic nervous system dysfunction. The aim of the study was to assess the predictive capacity of HRV markers, including HRF, for AF occurrence over an 18-year follow-up and to develop a predictive score for AF onset among the general population aged 65 at the study’s inception.

**Methods and results:**

The PROOF prospective cohort consisted of 1011 subjects aged 65 with no history of AF nor history of cardiovascular disease. A 24 h Holter-electrocardiogram was performed at baseline and HRV, from which HRV indices using temporal, frequency, and non-linear methods, and the percentage of inflection points (PIPs) were calculated. The PROOF cohort demonstrated a cumulative incidence of AF of 13.0% during a median follow-up of 17.8 years. Male gender, hypertension, decreased heart rate and α1, and increased premature atrial complex burden, PNN50, and PIP were independent predictors of AF occurrence. Subsequently, the PROOF-AF risk score was developed, ranging from 0 to 7, providing interesting predictive capacity [area under the curve (AUC) = 70.1%, negative predictive value = 92.0%, and accuracy = 72.0%]. The high-risk group (PROOF-AF score from 5 to 7) and the intermediate-risk group (PROOF-AF score from 2 to 4) exhibited a 16.8- and 5.4-fold higher risk, respectively, of developing AF.

**Conclusion:**

Heart rate fragmentation parameters, included in the PROOF-AF score, may be used to identify healthy individuals aged 65 who are at high risk of developing AF and assist population screening.

## Graphical Abstract

**Graphical Abstract:**
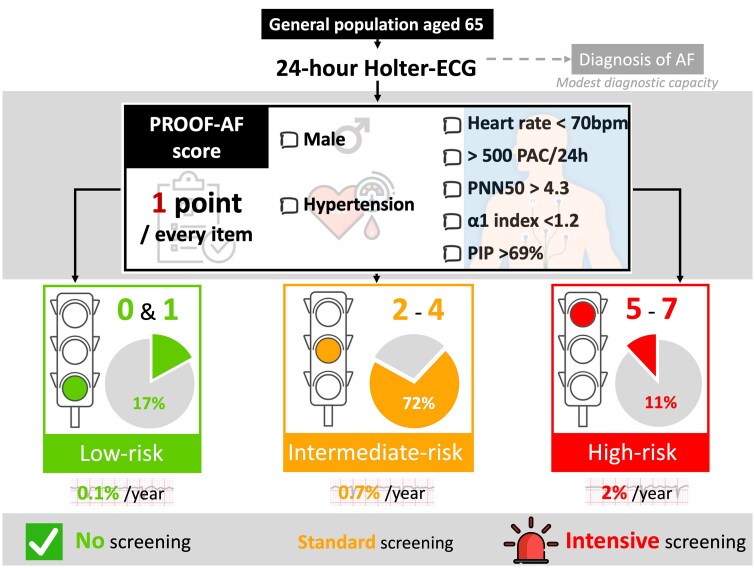

## Introduction

Atrial fibrillation (AF) affects approximately one in three individuals during their lifetime,^1^ and its prevalence is expected to rise with population ageing.^2^ While AF can often be asymptomatic, resulting in delayed diagnosis, it can lead to complications, such as ischaemic stroke, heart failure, and cognitive decline.^2^ Therefore, screening for AF is crucial to prevent these adverse outcomes. The European Society of Cardiology (ESC) guidelines recommend opportunistic screening for AF in the general population over 65 years using pulse taking or electrocardiogram (ECG) strip.^3^ However, the United States Preventive Services Task Force does not currently recommend AF screening in the general population over 50 due to the insufficient evidence supporting its superiority over standard care.^4^ The discrepancy between the different guidelines^5^ likely arises from the lack of a validated screening strategy and challenges associated with implementing widespread AF screening in the general population. To enhance AF screening, targeting a subpopulation at risk of developing AF within the general population could be advantageous. Assessment of abnormal autonomic nervous system (ANS) activity can serve as a screening tool, given its significant role in the initiation and maintenance of AF.^6,7^ Heart rate variability (HRV) analysis has been used for decades to evaluate ANS activity, but its usefulness as a predictor of AF has yielded conflicting data,^8–11^ certainly due to the fact that traditional short-term HRV metrics include heart rate fragmentation (HRF) and respiratory sinus arrhythmia, reflecting ANS breakdown and vagal tone, respectively.^12^ Nevertheless, newer metrics focusing specifically on HRF may provide a more accurate depiction of autonomic abnormalities and better predict AF in the general population.^13^ Therefore, we hypothesize that assessing HRF is valuable for stratifying the long-term risk of incident AF in individuals aged 65 within the general population.

The objective of this study is to assess the predictive capacity of HRF markers for the occurrence of AF over an 18-year follow-up in individuals aged 65 at the beginning of the study. Using these parameters, we aim to develop a predictive score specifically tailored to the general population aged 65 for predicting AF occurrence.

## Methods

### Study population

The PROOF study (NCT00759304), also known as the PROgnostic indicator OF cardiovascular and cerebrovascular events study, is a prospective cohort study conducted in an elderly population.^14^ Its main objective is to assess the predictive capacity of features depicting the level of ANS activity in relation to cardiovascular events and mortality. Between September 2000 and December 2002, invitations to participate in the PROOF study were sent to all individuals aged 65 and residing in Saint-Étienne, France. The invitations were sent out exhaustively, aiming to include as many eligible individuals as possible from the targeted population. Individuals with a history of AF, myocardial infarction, stroke, heart failure, or those who had a cardiac implantable electronic device were excluded from participation. Additionally, individuals with chronic diseases associated with a life expectancy of <5 years and those residing in institutions were also not included in the PROOF cohort. Out of the 3983 individuals contacted, 1011 eligible participants were initially included in the prospective cohort, representing 25.38% of the target population. The median follow-up was 17.8 years [interquartile range (IQR): 16.0–18.5 years]. During the follow-up period, 190 participants withdrew their consent, primarily within the first 3 years of the follow-up (*n* = 186, 97.9%). Additionally, 109 participants were excluded from the long-term follow-up due to meeting the exclusion criteria, such as loss of autonomy and institutionalization. No cases of loss to follow-up were reported.

The PROOF study received ethical approval from local and regional ethics committees (IRB-IEC, CCPRB). Data collection was authorized by the National Committee for Information and Liberty. All participants provided written informed consent before their involvement in the study.

### Heart rate variability assessment

The assessment of the clinical covariates is reported in the Supplementary material online. An increased number of premature atrial contractions (PACs) was defined as more than 500 per 24 h.^15^ The assessment of ANS activity was conducted at the time of inclusion using HRV parameters derived from a 24 h Holter ECG recording (StrataScan 563, Del Mar Avionics, Irvine, CA, USA). The time period between two consecutive R-wave peaks (RR intervals), which represent the time intervals between consecutive normal-to-normal (NN) heartbeats, were extracted from the 24 h ECG recordings. Artefacts and premature beats were corrected using the spleen cubic interpolation method as recommended.^16^ Various HRV indices were then calculated based on the ESC and ACC/AHA guidelines.^16^ A custom HRV analysis software^17^ was used for these calculations. Mean heart rate and the following HRV features were calculated:

- HRV time- and frequency-domain indices, as described in the Supplementary material online.
- In addition to traditional HRV metrics, novel HRV metrics were also assessed. These included the percentage of inflection point (PIP) in the NN interval time series, which served as a marker of HRF.^13^ Non-linear dynamical indices, such as α1,^13^ derived from short-term detrended fluctuation analysis, as well as acceleration (AC) and deceleration capacity (DC)^18^ were also calculated.

### Ascertainment of incident atrial fibrillation

The occurrence of incident AF cases was prospectively ascertained in accordance with the definition provided by international guidelines.^3^ As part of the ongoing follow-up of the PROOF cohort, a 24 h Holter ECG was systematically conducted at the time of participant inclusion, and repeated at 3, 6, and 12 years after inclusion. During clinical check-ups, any suspected AF cases were further investigated by performing a standard 12-lead ECG recording or a single-lead ECG tracing of 30 s to assess the heart rhythm with no discernible repeating *P* waves and irregular RR intervals. In cases where AF diagnosis was established by the general practitioner, AF tracings were collected to confirm the diagnosis. These rigorous diagnostic measures were employed to accurately identify incident cases of AF in the PROOF cohort.

### Risk scores

The methods describing the selection of the risk score are reported in the Supplementary material online. After applying these selection criteria, we compared the PROOF-AF score, which we developed, with five other risk scores for AF occurrence in the community. These included the CHARGE-AF,^19^ C_2_HEST,^20^ FHS,^21^ WHS,^22^ and JMC^23^ scores. By comparing the performance and utility of the PROOF-AF score with these established risk scores, we aimed to assess the effectiveness and applicability of our novel score in predicting AF occurrence in the general population.

### Statistical analysis

Continuous variables were presented as mean ± standard deviation, while median and IQR were used as appropriate. Categorical variables were expressed as total numbers and percentages. Except for increased burden of PACs (>500 PAC a day is commonly accepted^24^), no threshold for the HRV metrics was found in the literature. To determine the threshold values for the HRV metrics in the general population aged 65 regarding AF onset, receiver operating characteristic (ROC) analysis was conducted. This analysis aimed to establish the threshold that yielded the optimal sensitivity–specificity ratio for each HRV metric in predicting AF occurrence during the long-term follow-up in the PROOF prospective cohort. Supplementary material online, *Figure S1* provides additional details on these thresholds. For the survival analysis, participants who were lost to follow-up were censored at the time of their last follow-up assessment. The Kaplan–Meier method was used to estimate the time to event for AF occurrence, and the univariate log-rank test was employed to compare between groups. Hazard ratios (HRs) and corresponding 95% confidence intervals (CIs) were calculated. A forward Wald stepwise selection algorithm was applied to construct the multivariate Cox model, with covariates having a *P*-value <0.10 in the univariate analysis retained in the final model. Based on the results of the multivariate analysis, a risk score for AF occurrence in the PROOF prospective cohort during the long-term follow-up was developed, considering the effect size (HR) of each independent predictor. Independent predictors were defined as variables with a *P*-value <0.05 in the multivariate analysis. An internal validation of our predictive model was made by bootstrap analysis of 1000 samples.^25^ In the bootstrap procedure, repeated samples of the same number of observations as the original database were randomly selected with replacement from the original set of observations. The predictive capacity of the developed PROOF-AF score and other available risk scores was evaluated using ROC methodology. All statistical tests used a two-sided Type I error of 5%. The statistical analysis was performed using R software for Windows version 4.2.1 (R Project for Statistical Computing, Vienna, Austria).

## Results

### Characteristics of the study population

From September 2000 to December 2002, a total of 1011 individuals aged 65 were included in the PROOF prospective cohort. The baseline characteristics of the PROOF cohort are summarized in *Table 1*. Most participants were female (60.2%), and a significant proportion had a history of tobacco use (36.1%), hypertension (36.1%), dyslipidaemia (37.7%), obesity (10.7%), thyroid disorders (14.8%), and severe obstructive sleep apnea (OSA) (15.8%). Diabetes mellitus was present in 5.6% of the participants. *Table 2* provides the descriptive features of HRV analysis, including time-domain, frequency-domain, and novel HRV-derived metrics.

**Table 1:** Baseline characteristics of the patients included in the prospective PROOF cohort

	All (*n* = 1011)	
Clinical features		
Age	65.1	65.0–66.1
Male gender	402	39.8%
Diabetes mellitus	57	5.6%
Hypertension	365	36.1%
Dyslipidaemia	381	37.7%
Thyroid disorders	150	14.8%
COPD	51	4.6%
History of tabacco use	364	36.1%
Alcohol use	452	45.0%
Obesity	108	10.7%
Severe OSA	134	15.8%
24 h Holter ECG		
Average heart rate (b.p.m.)	71	66–78
Decreased heart rate	518	51.2%
PAC burden (per hour)	2	1–6
Increased PAC burden	92	9.1%

Continuous variables are presented as mean ± standard deviation, while median-IQR was used as appropriate. Categorical variables were expressed as total numbers and percentages.

COPD, chronic obstructive pulmonary disease; HF, high frequency; HRV, heart rate variability; LF, low frequency; OSA, obstructive sleep apnoea; PAC, premature atrial contraction.

**Table 2:** Baseline cardiac autonomic function of the patients included in the prospective PROOF cohort derived from parameters of heart rate variability

	All (*n* = 1011)	
Time-domain HRV analysis		
SDNN (ms)	128	108–154
SDANN (ms)	117	97–141
RMSSD (ms)	23.5	18.6–30.0
Increased RMSSD	302	29.9%
PNN50 (%)	3.8	1.6–7.6
Increased PNN50	454	44.9%
Frequency-domain HRV analysis		
Total power (ms^2^)	8400	4844–15 075
Total power (log)	3.9	3.7–4.2
VLF (ms^2^)	1208	746–1757
VLF (log)	3.1	2.9–3.2
Normalized VLF (%)	14.2	9.0–21.3
LF (ms^2^)	399	242–675
LF (log)	2.6	2.4–2.8
Normalized LF (%)	5.0	2.9–8.4
HF (ms^2^)	108	64–192
HF (log)	2.0	1.8–2.3
Normalized HF (%)	1.3	0.8–2.3
LF/HF	5.3	3.9–7.4
Increased LF/HF (roc)	480	47.9%
Novel HRV-derived metrics		
PIP (%)	72.0	±6.3
Increased PIP	681	67.8%
α1	1.26	1.15–1.39
Decreased α1	682	67.9%
Sample entropy (SampEn)	1.09	1.00–1.19
Acceleration capacity (ms)	7.1	5.9–8.5
Increased acceleration capacity	420	41.5%
Deceleration capacity (ms)	6.7	5.6–8.0
Decreased deceleration capacity	675	66.8%

Continuous variables are presented as mean ± standard deviation, while median-IQR was used as appropriate. Categorical variables were expressed as total numbers and percentages.

Cut-off values of HRV metrics correspond to the value that yields the optimal sensitivity–specificity ratio using ROC analysis regarding AF occurrence. Increased LF/HF: LF/HF > 5.4; increased PIP: PIP > 69%; decreased α1: α1 < 1.2; increased acceleration capacity: AC > 5.5; decreased deceleration capacity: DC > 7.5.

HF, high frequency; HRV, heart rate variability; LF, low frequency; OSA, obstructive sleep apnoea; PAC, premature atrial contraction; PIPs, percentage of inflection points; PNN50, percentage of normal-to-normal RR (NN) intervals that differ by >50 ms; SDANN, standard deviation of the averages of NN intervals; RMSSD, root mean square differences of successive NN intervals; SDNN, standard deviation of NN intervals; VLF, very low frequency.

During a median follow-up of 17.8 years (IQR: 16.0–18.5), clinical AF was diagnosed in 123 participants. The cumulative incidence of AF at 5, 10, 15, and the median 17.8-year follow-up was 4.1% (95% CI: 2.9–5.3), 7.0% (95% CI: 5.4–8.6), 10.7% (95% CI: 8.7–12.6), and 13.0% (95% CI: 10.8–15.2), respectively, as depicted in Supplementary material online, *Figure S2*. The overall incident rate of AF in the entire PROOF cohort population during the median 17.8-year follow-up was 0.77 per 100 persons/year.

### Predictors of atrial fibrillation occurrence

In the univariate analyses presented in *Table 3*, several clinical parameters were found to be associated with AF onset during the 18-year follow-up, such as male gender (HR = 1.74, 95% CI: 1.22–2.48), hypertension (HR = 1.78, 95% CI: 1.25–2.53), and obesity (HR = 1.88, 95% CI: 1.22–2.48). Tobacco use, severe obstructive sleep apnoea, and sedentary lifestyle showed a trend of association with incident AF (*P*-values of 0.06, 0.09, and 0.07, respectively). Heart rate below 70 b.p.m. (HR = 1.70, 95% CI: 1.17–1.46), increased PAC burden (HR = 2.11, 95% CI: 1.31–3.41), increased RMSSD (HR = 1.90, 95% CI: 1.33–2.71), PNN50 (HR = 1.84, 95% CI: 1.28–2.64), PIP over 69% (HR = 2.20, 95% CI: 1.40–3.47), decreased ration of low frequency to high frequency (LF/HF) (HR = 2.02, 95% CI: 1.38–2.96), α1 index (HR = 2.19, 95% CI: 1.54–3.12), AC (HR = 1.52, 95% CI: 1.04–2.22), DC (HR = 2.02, 95% CI: 1.30–3.09), and SD1/SD2 (HR = 2.40, 95% CI: 1.23–3.92) were metrics derived from 24 h Holter ECG significantly associated with AF occurrence during the 18-year follow-up in univariate analysis. Interestingly, daytime HRV parameters are consistent with those derived from 24 h Holter monitoring, unlike the parameters obtained from night-time recordings (Supplementary material online, *Figure S3*). In the multivariate analysis, after adjusting for other variables, seven independent parameters remained significantly associated with AF occurrence during the 18-year follow-up (*Table 3*): male gender, hypertension, decreased heart rate, increased PAC burden, increased PNN50, increased PIP, and decreased α1 index. It is important to note that no interaction was found between sex and the other independent predictors of AF occurrence (Supplementary material online, *Figure S4*).

**Table 3:** Uni- and multivariate Cox proportional hazard models for incident atrial fibrillation during a 18-year follow-up

	Unadjusted			Adjusted		
	HR	95% CI	*P*-value	HR	95% CI	*P*-value
**Clinical features**						
Male gender	1.74	(1.22–2.48)	<0.01	**1.70**	**(1.17–2.47)**	**<0.01**
Diabetes mellitus	1.58	(0.80–3.11)	0.19			
Hypertension	1.78	(1.25–2.53)	<0.01	**1.44**	**(1.05–2.06)**	**0.04**
Dyslipidaemia	0.95	(0.66–1.37)	0.79			
Thyroid disorders	0.90	(0.54–1.51)	0.69			
COPD	0.84	(0.34–2.06)	0.70			
Tabacco use	1.41	(0.99–2.01)	0.06			
Alcohol use	1.11	(0.78–1.59)	0.56			
*Obesity*	1.88	(1.19–2.99)	<0.01	–	–	–
Severe OSA	1.50	(0.93–2.43)	0.09			
Physical inactivity	0.93	(0.85–1.01)	0.07			
**24 h Holter ECG**						
Decreased heart rate	1.70	(1.17–1.46)	<0.01	**1.74**	**(1.16–2.59)**	**<0.01**
Increased PAC burden	2.11	(1.31–3.41)	<0.01	**1.94**	**(1.19–3.16)**	**0.01**
**Time-domain HRV analysis**						
SDNN	0.99	(0.99–1.00)	0.64			
SDANN	1.00	(0.99–1.00)	0.31			
*Increased RMSSD*	1.90	(1.33–2.71)	<0.01	–	–	–
Increased PNN50	1.84	(1.28–2.64)	<0.01	**1.59**	**(1.04–2.43)**	**0.03**
**Frequency-domain HRV analysis**						
Total power (log)	1.14	(0.69–1.91)	0.61			
VLF (log)	1.32	(0.69–2.55)	0.41			
Normalized VLF	0.83	(0.10–6.57)	0.86			
LF (log)	0.91	(0.54–1.54)	0.72			
Normalized LF	0.25	(0.01–16.80)	0.52			
HF (log)	1.25	(0.77–2.02)	0.37			
Normalized HF	39.44	(0.00–528.00)	0.43			
*Decreased LF/HF*	2.02	(1.38–2.96)	<0.01	–	–	–
**Novel HRV-derived metrics**						
Increased PIP	2.20	(1.40–3.47)	<0.01	**2.74**	**(1.69–4.47)**	**<0.01**
Decreased α1	2.19	(1.54–3.12)	<0.01	**1.67**	**(1.11–2.51)**	**0.02**
SampEn	0.86	(0.26–2.86)	0.80			
*Decreased acceleration capacity*	1.52	(1.04–2.22)	0.03	–	–	–
*Decreased deceleration capacity*	2.02	(1.30–3.09)	<0.01	–	–	–
*SD1/SD2*	2.40	(1.23–3.92)	<0.01	–	–	–

Data in italic: covariates with a *P*-value of <0.05 in the univariate analysis and included in the multivariate Cox model. Data in bold: independent risk factors with a *P*-value of <0.05 using multivariate analysis. Cut-off values of HRV metrics correspond to the value that yields the optimal sensitivity–specificity ratio using ROC analysis regarding AF occurrence. Increased LF/HF: LF/HF > 5.4; increased PIP: PIP > 69%; decreased α1: α1 < 1.2; increased acceleration capacity: AC > 5.5; decreased deceleration capacity: DC > 7.5.

COPD, chronic obstructive pulmonary disease; HF, high frequency; HR, hazard ratio; HRV, heart rate variability; LF, low frequency; OSA, obstructive sleep apnoea; PAC, premature atrial contraction; PIP, percentage of inflection points; PNN50, percentage of normal-to-normal RR (NN) intervals that differ by >50 ms; SDANN, standard deviation of the averages of NN intervals; RMSSD, root mean square differences of successive NN intervals; SDNN, standard deviation of NN intervals; VLF, very low frequency; 95% CI, 95% confidence interval.

### Risk score

**Figure 1:**
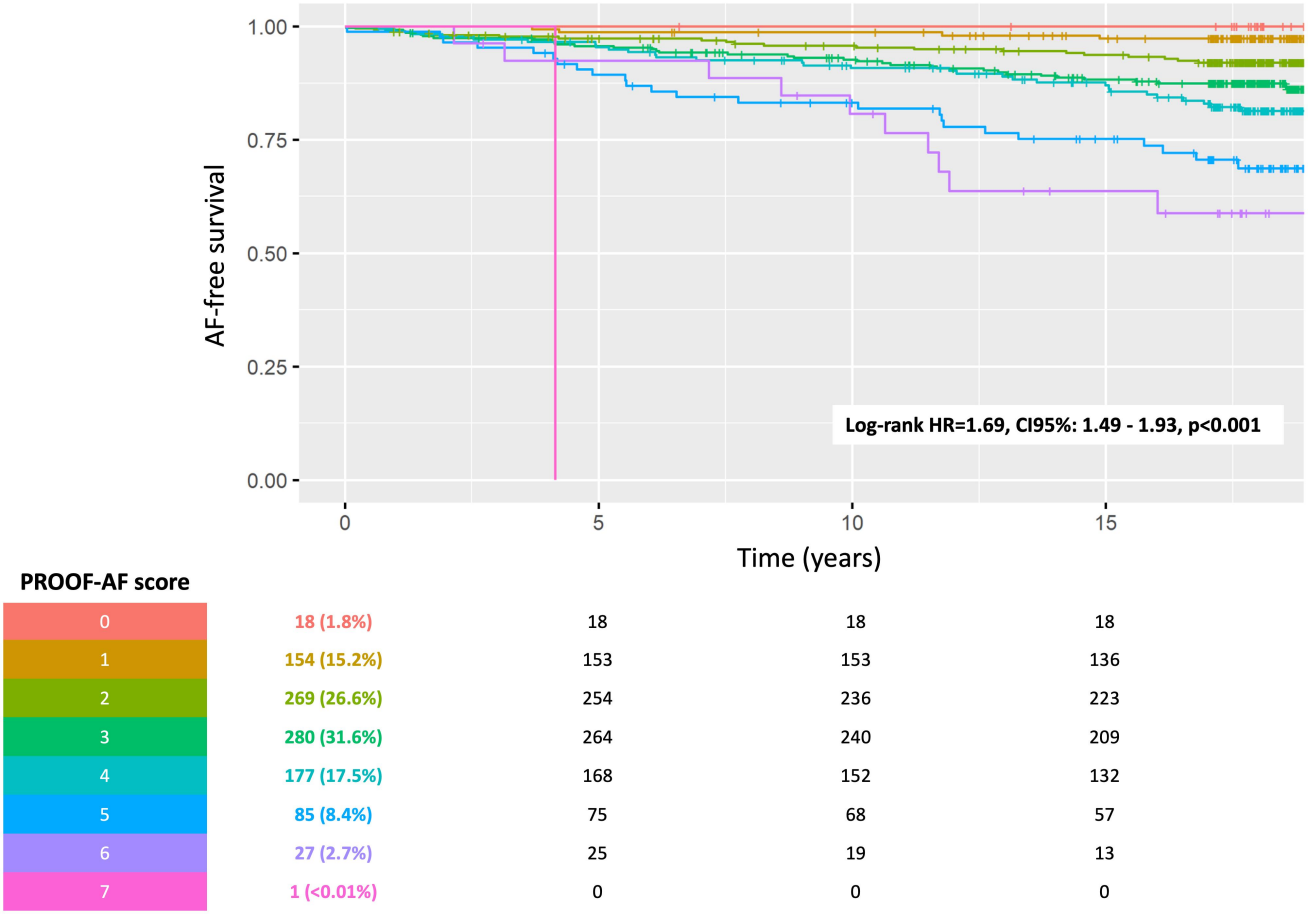
Kaplan–Meier analysis of atrial fibrillation according to the PROOF-AF score within the long-term follow-up. The table in the bottom part of the figure reports the number of individuals at risk (percentage) at baseline, 5-, 10-, and 15-year follow-up

**Figure 2:**
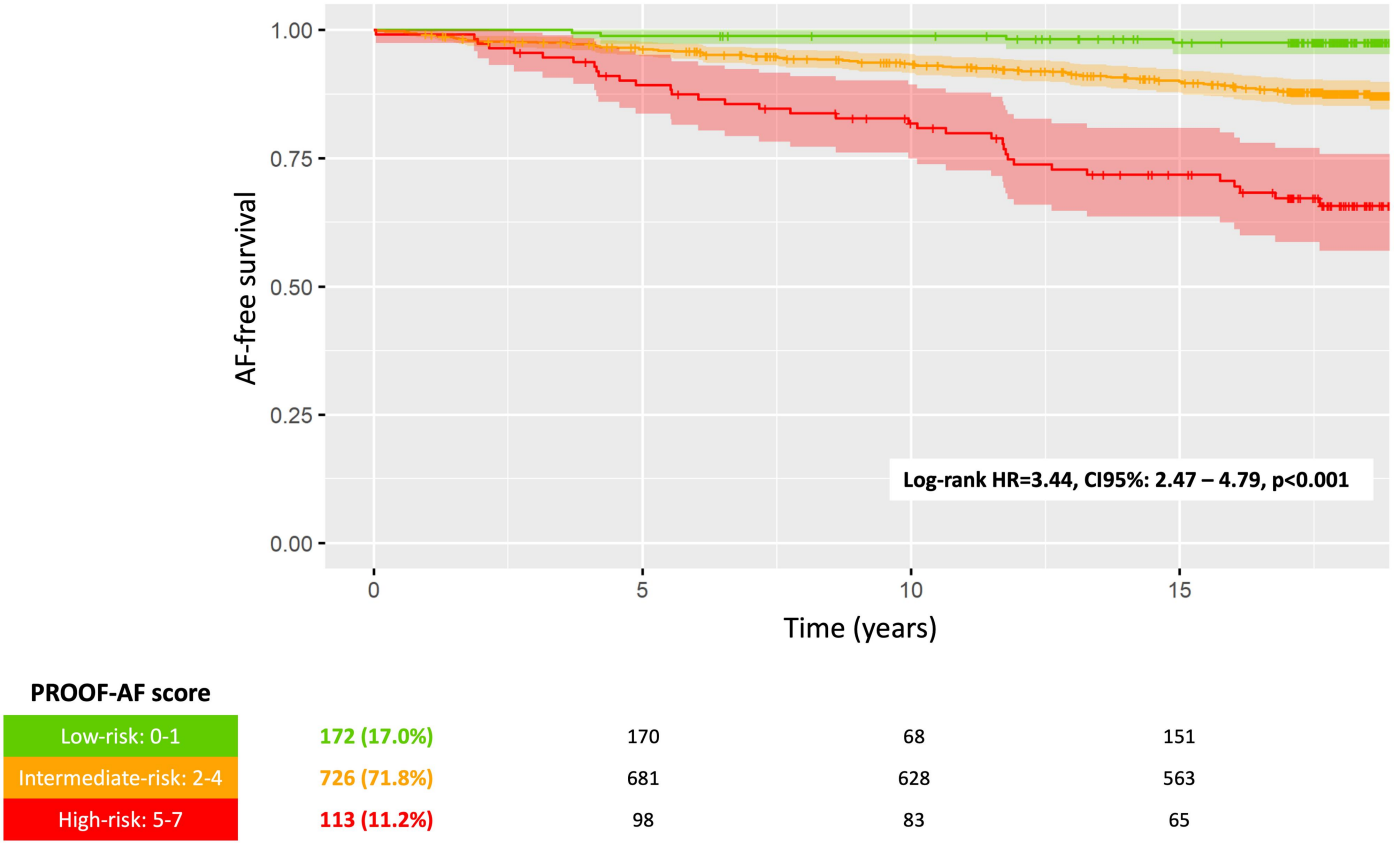
Kaplan–Meier analysis of atrial fibrillation in the low-, intermediate-, and high-risk group during the long-term follow-up. The table in the bottom part of the figure reports the number of individuals at risk (percentage) at baseline, 5-, 10-, and 15-year follow-up.

Based on the results of the multivariate analysis results, a risk score for AF occurrence during the long-term follow-up in the PROOF prospective cohort was developed, considering the effect size of each independent predictor. The PROOF-AF risk score ranged from 0 to 7 points and was based on the presence of specific predictors, giving 1 point if an independent predictor was present at the inclusion: male sex, hypertension, heart rate below 70 b.p.m., increased PAC burden, PNN50 >4.3, PIP >69%, α1 index below 1.2. The baseline PROOF cohort was divided into different risk groups based on their scores: 18 individuals (1.8%) had a score of 0, 154 individuals (15.2%) had a score of 1, 269 individuals had a score of 2 (26.6%), 280 individuals had a score of 3 (27.7%), 177 scoring 4 (17.5%), 85 (8.4%) had a score 5, 27 (2.7%) scoring 6, and 1 individual had a top score of 7. The estimated incidence of AF during the median 17.8-year follow-up was estimated at 2.7% (0.01–5.3), 8.0% (4.6–11.3), 13.8% (9.1–18.4), 18.7% (12.3–24.5), 31.3% (19.8–41.1), 41.2% (17.6–58.0) for a PROOF-AF risk score from 1 to 7, respectively (*Figure 1*). The PROOF-AF score had a positive and negative predictive value of 22.4 and 92.0%, respectively (Supplementary material online, *Figure S5*), an accuracy of 72.0%, and an area under the curve of 70.1% (95% CI: 65.4–74.7). The entire initial PROOF cohort was then divided into three different groups (*Figure 2*): a group with a low risk of AF occurrence, scoring 0 and 1; a subgroup with a high risk of AF onset, with a score ≥6; and an intermediate-risk subpopulation in between.

The low-risk group consisted of 172 participants (17.0%) at baseline, with an estimated incidence of AF at 2.4% (0.0–4.7), and an incident rate of AF reported as 0.13 per 100 persons/year. The high-risk group (PROOF-AF scoring from 5 to 7) included 113 participants (11.2%) at baseline, with an estimated incidence of AF at 34.3% (24.2–43.1) during the follow-up period, and an incident rate of AF reported as 1.93 per 100 persons/year. The intermediate-risk group, with a PROOF-AF score from 2 to 4, comprised 726 individuals (71.8%), with an estimated AF incidence of 12.9% (10.2–15.5), and an incident rate of 0.72 per 100 persons/year. Compared with the low-risk group, patients in the intermediate-risk group had a 5.4-fold higher risk of developing AF (95% CI: 1.99–14.79, *P* < 0.01), while the high-risk group showed a 16.8-fold increased risk of AF occurrence during the long-term follow-up (95% CI: 5.97–47.27, *P* < 0.01).

### Internal validation of the risk score and comparison with established risk scores

**Figure 3:**
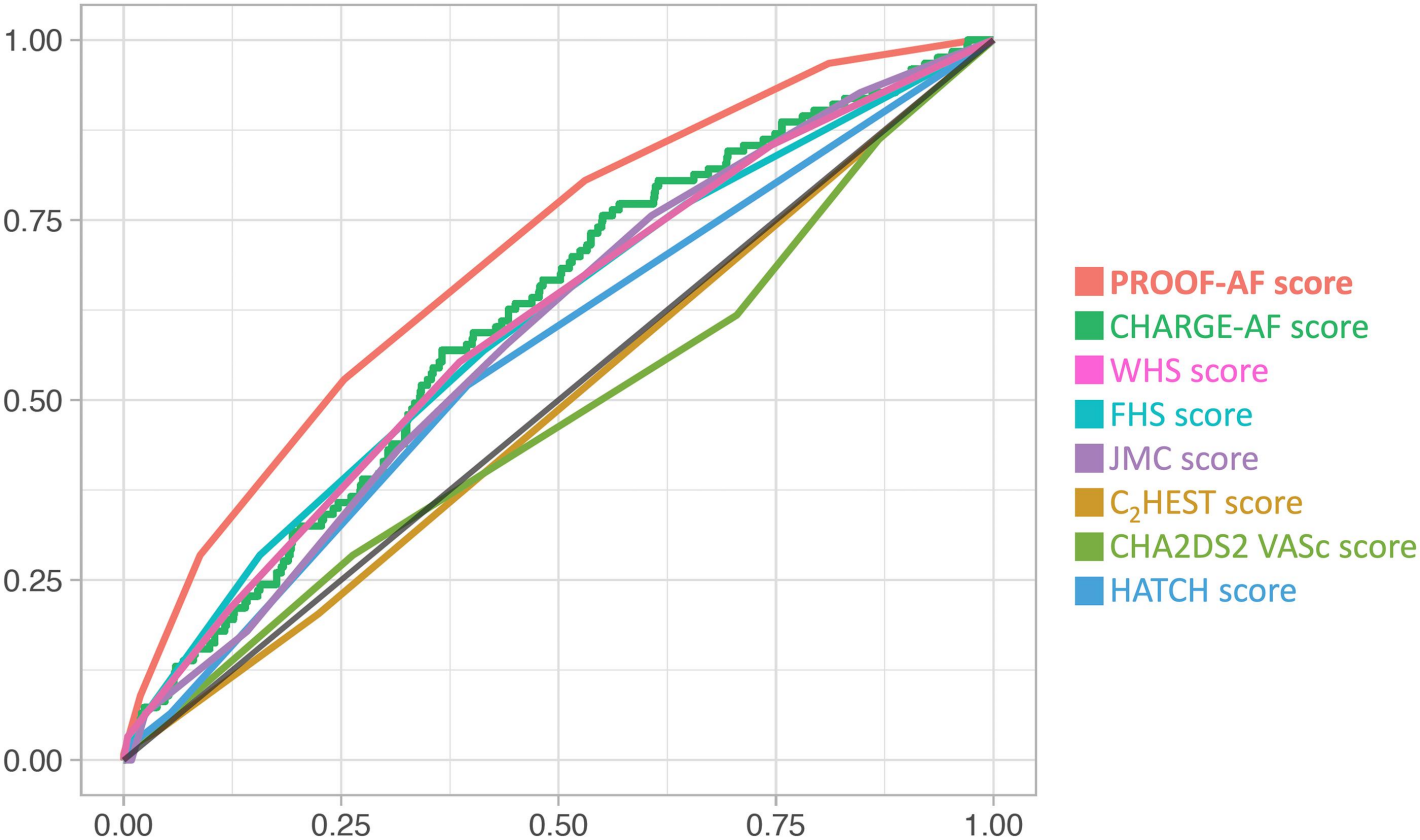
Receiver operating characteristic curve of the PROOF-AF risk score and the other available risk scores for predicting atrial fibrillation occurrence during a long-term follow-up in the general population aged 65 at baseline.

The PROOF-AF risk score was internally validated using bootstrap HR estimation. The internally validated HR for the PROOF-AF risk score was 1.69 (95% CI: 1.51–1.94, *P* < 0.001). Furthermore, the predictive capacity of a PROOF-AF score was also internally validated. Compared with the low-risk group, patients in the intermediate-risk group had a 5.4-fold higher risk of developing AF (95% CI: 2.46–25.23, *P* < 0.01), while the high-risk group showed a 16.8-fold increased risk of AF occurrence during the long-term follow-up (95% CI: 7.42–78.65, *P* < 0.001). Comparing the predictive capacities of available risk scores for AF occurrence during long-term follow-up in the general population, the PROOF-AF score demonstrated superior performance (*Figure 3*). The *c*-statistic for the PROOF-AF score was 0.701 (95% CI: 0.653–0.748). In comparison, the CHARGE-AF score had a *c*-statistic of 0.614 (95% CI: 0.563–0.665), the WHS score had a *c*-statistic of 0.604 (95% CI: 0.551–0.657), the FHS score had a *c*-statistic of 0.603 (95% CI: 0.549–0.657), the JMC score had a *c*-statistic of 0.591 (95% CI: 0.540–0.642), the C_2_HEST score had a *c*-statistic of 0.490 (95% CI: 0.436–0.545), and the CHA_2_DS_2_ VAS_c_ score had a *c*-statistic of 0.481 (95% CI: 0.424–0.538).

## Discussion

### Main findings

During the median 17.8-year follow-up, 123 out of the 1011 healthy participants aged 65 at baseline developed AF, resulting in a cumulative incidence of 13.0%. Male gender, hypertension, heart rate below 70 b.p.m., increased PAC burden, PNN50 and PIP, and decreased α1 index were identified as independent predictors of AF occurrence during the long-term follow-up. Based on these predictors, the PROOF-AF risk score was developed and demonstrated the ability to discriminate different levels of risk for AF occurrence among the PROOF cohort. Specifically, individuals at high risk (PROOF-AF score >5) were found to have a 16.8-fold higher risk of developing AF during the follow-up period. The PROOF-AF score showed superior performance compared with other available risk scores in predicting incident AF during a long-term follow-up in the PROOF cohort.

### Heart rate fragmentation as a novel parameter to assess autonomic nervous system abnormal activity and incident atrial fibrillation

The ANS plays a key role in the initiation and maintenance of AF. Both the sympathetic and parasympathetic branches of the ANS contribute to the development of triggers and atrial substrate for AF.^6^ Cholinergic activation leads to a spatially heterogeneous decrease in the action potential of atrial cardiomyocytes by activating *I*_K,Ach_ current. Adrenergic activation, on the other hand, is responsible for early and delayed afterdepolarizations and contributes to atrial structural changes through neurohormonal activation and extracellular matrix remodelling.^26^ Assessing abnormalities in ANS activity can be a relevant strategy for screening individuals at risk for AF occurrence and preventing its complications.^27^ Heart rate variability assessment is a non-invasive and validated method for assessing ANS activity by analysing the variability of the RR intervals on an ECG tracing^28^ of short or long duration. However, studies investigating the association between HRV parameters and incident AF during long-term follow-up have provided conflicting results. While data derived from the Framingham cohort suggest the lack of an independent association between HRV metrics and incident AF,^29^ several studies highlighted the association between increased HRV parameters^9,10^ and AF, and others the contrary.^8,11^ There are several possible explanations for these discrepancies. First, the studies use different sources and durations of ECG recordings, ranging from 2 min^10^ to 24 h.^9^ Second, the interpretation of short-term HRV metrics may vary, and recent evidence suggests that HRF, a marker of abnormal global ANS activity abnormality,^13,30^ can influence the interpretation of short-term HRV parameters in specific populations.^31,32^ In our study, we aimed to highlight the independent association between the autonomic phenotype of healthy individuals aged 65 and the risk for AF occurrence. We found that several HRV parameters, such as PNN50, were independent predictors of AF new onset contradicting previous population-based studies that reported an association between decreased PNN50 and increased risk of cardiovascular disease and mortality. Traditional HRV parameters, such as PNN50, RMSSD, and the LF/HF ratio, are typically considered indicators of parasympathetic activity in a healthy and young population. However, our study suggests that these parameters, along with HRF parameters, can predict AF onset in individuals aged 65 years and older, indicating that the traditional HRV parameters may reflect a breakdown of global ANS activity rather than parasympathetic activity (Supplementary material online, *Figure S6*). The PIP parameter, which represents abnormal global ANS activity and is independent of the heart rate and daytime,^13^ emerged as a relevant marker of HRF. Additionally, a decreased α1 index, derived from detrended fluctuation analysis, reflects the correlation properties of short-term time series in a spiky shape and may serve as an additional marker of HRF.^33^ The findings of our study, along with other published data^34,35^ demonstrating the independent association between HRF and incident AF, may represent an initial step in understanding the link between ANS dysfunction and other cardiovascular events such as heart failure and myocardial infarction.^33,36,37^ Contrary to what is reported in the literature,^38^ decreased AC and DC, calculated from the 24 h Holter ECG and designed to specifically assess vagal tone, were associated with incident AF in univariate analysis but lost their significance in multivariate analysis. It is also important to note that although the various HRV indices assess the activity of the ANS, it cannot be ruled out that HRV may also reflect intrinsic cardiac conduction abnormalities.

### Predicting atrial fibrillation occurrence in the general population

The development of adequate screening tools for AF in the general population is a challenge that needs to meet certain prerequisites established by the World Health Organization.^39^ Currently available screening tools have limitations in terms of diagnostic performance and acceptance by the target population.^5^ Randomized clinical trials, such as STROKE-STOP^40^ and LOOP,^41^ have shown either no significant benefit or only mild benefit of a screening strategy for AF in a general population. The method of screening the community for AF remains a major challenge. Short-term diagnostic tools, although inexpensive, have a low diagnostic performance, such as 2% detection rate reported in the VITAL-AF study.^42^ On the other hand, connected devices offer better diagnostic performance but have drawbacks in terms of cost-effectiveness^43^ and the lack of validated therapeutic management for subclinical episodes of AF.^41^ Therefore, choosing the target population that could benefit the most from AF screening seems crucial to improve diagnostic performance, feasibility, and acceptability of the diagnostic strategy. In the PROOF-AF study, we propose a different approach by preselecting a high-risk population for long-term AF occurrence using a low-cost and commonly used diagnostic tool in clinical practice, the 24 h Holter ECG. Other predictive risk factors have been suggested to identify a population at risk for AF,^19–23^ but they come with various limitations, such as the need for sophisticated calculation tools^19^ or additional tests like echocardiography.^44^ In addition, the PROOF-AF risk score demonstrated superior performance compared with other predictive scores for long-term AF occurrence.

### Clinical perspectives

The PROOF-AF study introduces a predictive risk score for long-term AF occurrence based on data obtained from a 24 h Holter ECG in patients aged 65 and considering the patient gender and history of hypertension. The PROOF-AF score consists of seven items and can be calculated without the need for external and sophisticated tools in routine clinical practice. One unique aspect of the study is that all participants included in the PROOF-AF cohort were aged 65 at the time of enrolment. Therefore, age is not considered in the predictive score, allowing for risk stratification within a population of the same age. The study not only provides a new predictive score for long-term AF risk assessment but also proposes an innovative diagnostic strategy for AF screening in the general population (*Graphical abstract*). The proposed methodology suggests performing a 24 h Holter ECG on the entire population aged 65, aiming to achieve two objectives: (i) screening for AF directly, even though the diagnostic performance may be modest according to ESC guidelines^3^ and (ii) establishing a level of risk for long-term incident AF. For individuals identified as high risk, an intensified screening strategy for AF could be implemented, considering their estimated 30% risk for AF occurrence within the next 15 years. This intensive screening could be performed either through ECG and Holter ECG recordings or by analysing long-term heart rate using wearable devices. On the other hand, individuals classified as low risk would not beneficiate from the standard screening due to an incident rate of 0.1%/year. The main part of the population included in the intermediate-risk group would beneficiated from the strategy recommended by the ESC guidelines, including opportunistic screening between 65 and 75 years of age and systemic screening beyond that age, considering the moderate incident AF rate of 0.7%/year. Before implementing this new diagnostic strategy, it is important to validate it through a large randomized controlled trial to assess its effectiveness in improving AF screening and preventing associated complications, such as stroke, heart failure, and cognitive decline.

### Study limitations

The PROOF-AF study has several limitations that need to be considered. Firstly, the study has a monocentric design. The inclusion and follow-up in a unique French city may prevent the extrapolation of the results to different populations and cultures. It would be beneficial to replicate the study in diverse settings to validate the findings across different populations. Although the long-term prospective design of the study is a strength, it is important to acknowledge that the systematic screening for AF was conducted as part of the PROOF study at specific intervals (3, 6, and 12 years) using a 24 h Holter ECG. More frequent screening intervals could have potentially improved the detection of AF cases. However, the study utilized routine clinical practice data to capture AF diagnoses, providing a pragmatic assessment of AF occurrence during the median 17.8-year follow-up. The PROOF-AF study did not include data derived from biomarkers, ECG, or echocardiogram parameters. This is a potential limitation of the study, as it is not possible to determine whether these parameters could have improved the predictive capacity of the PROOF-AF score. While the PROOF-AF score was developed based on the long-term follow-up of the PROOF prospective cohort, it still needs external validation in an independent cohort. Internal validation was performed using bootstrap analysis and comparison with existing scores, but the inclusion of an external cohort would strengthen the robustness and reliability of the PROOF-AF score before its implementation in clinical practice.

## Conclusions

Abnormal HRV, including increased PNN50, decreased α1 index, and increased PIP—a marker of HRF—are independent predictors of long-term incident AF in the PROOF study. These parameters, along with heart rate below 70 b.p.m., increased PAC burden, hypertension, and male gender, are integrated into the PROOF-AF score that can identify a high-risk population for AF among individuals aged 65. This risk score may be used to preselect individuals for a personalized and intensified AF screening.

## Lead author biography


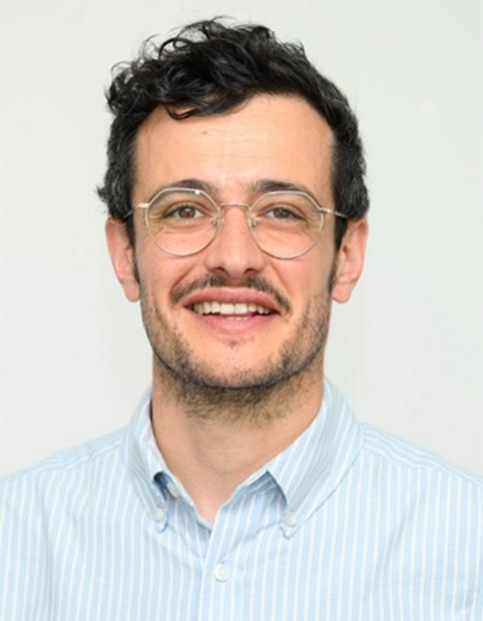


Jean-Baptiste Guichard is a clinician-researcher specializing in cardiac electrophysiology. His primary research focus is the characterization of atrial cardiopathy, both at the mechanistic level through translational research projects and in enhancing diagnostic and therapeutic management strategies. As part of this research, he investigates the impact of autonomic nervous system dysfunction on the development and progression of atrial cardiopathy and atrial fibrillation. After completing his initial medical training in France (Université de Saint-Étienne) and a PhD in Canada (Université de Montréal, Quebec), he is now a clinician-researcher at the arrhythmia unit of the Hospital Clínic de Barcelona, Universitat de Barcelona, in Catalonia, Spain.

## Supplementary Material

oeaf030_Supplementary_Data

## Data Availability

The data used in the PROOF-AF study and presented in this article are available from the corresponding author.
