# Supplementary material for: Assessing heart rate fragmentation to predict atrial fibrillation in the general population aged 65: the PROOF-AF study
Source: Eur Heart J Open. 2025 Mar 19;5(3):oeaf030. doi: 10.1093/ehjopen/oeaf030 (PMC12042749; doi:10.1093/ehjopen/oeaf030)
Supplement: oeaf030_Supplementary_Data [file oeaf030_supplementary_data.pdf]

Supplemental material

1. **Methods** 1
2. **Online Figures** 3

1. Methods

Clinical covariates

Clinical covariates were obtained from the baseline clinical data collected at the time of patient inclusion. Patients provided self-reported information regarding their history of tobacco use and alcohol consumption. Obesity was defined as a Body Mass Index (BMI) greater than 30, calculated as weight divided by height squared (kg/cm^2). Diabetes mellitus was defined as having a fasting glucose exceeding 126mg/dl or currently taking medication for diabetes. Hypertension was defined as meeting one of the following criteria: an average systolic blood pressure (BP) among three measurements exceeding 140mmHg, an average diastolic BP exceeding 90mmHg diastolic, or the use of antihypertensive drugs. Thyroid disorder was identified based on the use of medication for hypo- or hyperthyroidism, while dyslipidemia was determined using medication for lipid disorders. Severe obstructive sleep apnea was defined as an apnea hypopnea index (AHI) exceeding 30 per hour, as measured during the home sleep study. Physical activity was assessed using the Population Physical Activity Questionnaire (POPAQ), which relied on self-reported physical activity during the previous 7 days[15,16]. Physical inactivity was defined as having a metabolic equivalent of task (MET-h) less than 7.5 MET per week[17], which is equivalent to less than 30 minutes of moderate (3-5.9 MET) to vigorous (≥6 MET) physical activity per day, for five days per week.

Heart Rate Variability (HRV) assessment

The following classical HRV features were calculated:

- *HRV time-domain indices*. These included the percentage of NN intervals that differ by more than 50ms (PNN50), the root mean square differences of successive NN intervals (RMSSD), the standard deviation of NN intervals (SDNN), and the standard deviation of the averages of NN intervals calculated on consecutive five-minute periods (SDANN).
- *HRV frequency-domain indices*. Fourier transform was used to calculate total power, very low frequency (VLF: 0.003 to 0.04 Hz), low frequency (LF: 0.04 to 0.15 Hz), and high frequency (HF: 0.15 to 0.4 Hz) parameters. Raw, logarithmic, and relative values were computed for each parameter. Additionally, the LF/HF ratio was computed.

Risk scores

Through an exhaustive literature search conducted up to January 2023, we identified a total of 20 risk scores for AF prediction in general population. Out of these, 13 risk scores included a prospective follow-up component. To select the most relevant risk scores for our study, we filtered the validated risk scores using clinical data. We excluded risk scores that were based solely on echocardiographic features, genetic status, or other variables that were not collected at the time of inclusion in the PROOF study. We also excluded studies that provided risk prediction models that were not suitable for daily clinical practice.

2. Online Figures

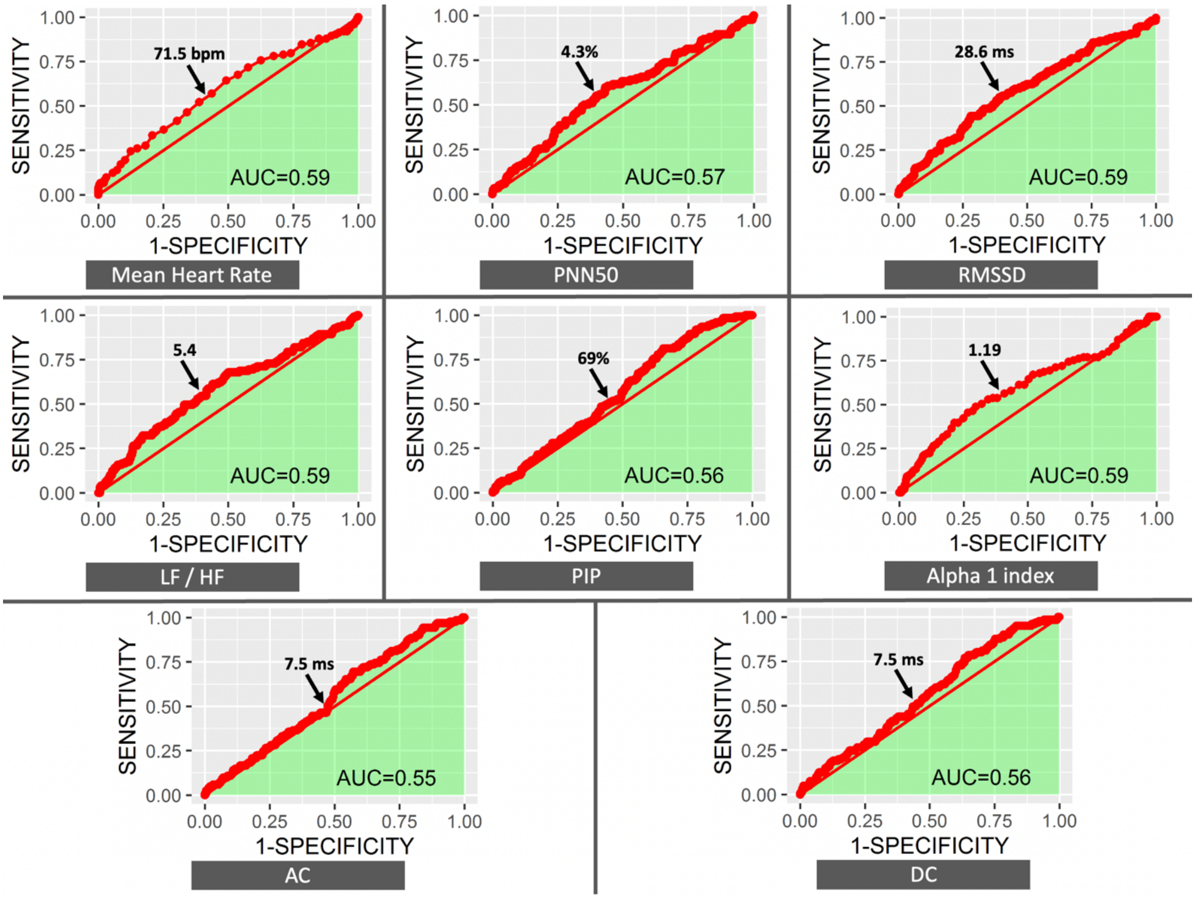

Online Figure 1. Graphical representation of the Receiver Operating Characteristic (ROC) analysis performed to determine the threshold values for various metrics of Heart Rate Variability (HRV) derived from 24-hour Holter-ECG in relation to the occurrence of AF occurrence in the PROOF prospective cohort.

For each HRV metric that showed a significant association with AF occurrence in the univariate analysi, a ROC analysis was systematically performed to establish a cutoff value and discretize the quantitative variable.

AUC: area under the curve; HF: high frequency; LF: low frequency; PIP: percentage of inflection points; PNN50: percentage of normal-to-normal RR (NN) intervals that differ by more than 50 ms; RMSSD: root mean square differences of successive NN intervals.

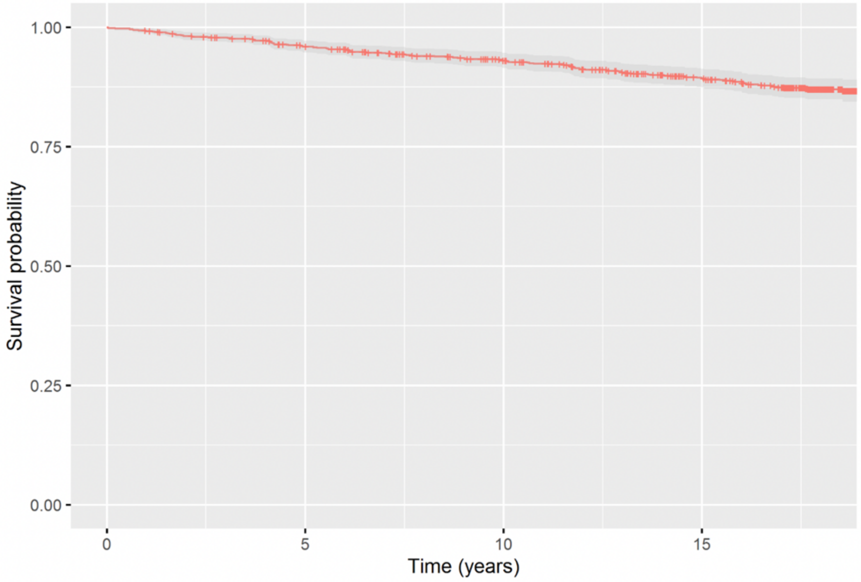

Number at risk	1011	949	877	779	539
Cumultaive incidence of AF	-	4.1% (2.9-5.3)	7.0% (5.4-8.6)	10.7% (8.7-12.6)	13.0% (10.8-15.2)

Online Figure 2. Kaplan-Meier analysis of atrial fibrillation within the median 17.8-year follow-up. The table in the bottom part of the figure reports the number of individuals at risk and the cumulative incidence of AF (95%CI) at baseline, 5, 10, 15, and 17.8-year follow-up.

	24-hour (n=1011)		Daytime (n=1011)		Nighttime (n=1011)	
Time-domain HRV analysis						
RMSSD (ms)	23.5	18.6 - 30.0	20.5	16.3 - 26.6	26.4	20.5 - 36.0
pNN50 (%)	3.8	1.6 -7.6	2.4	1.1 - 5.5	5.1	2.0 - 11.6
Frequence-domain HRV analysis						
LF / HF	5.3	3.9 - 7.4	4.1	2.8 - 6.0	3.4	2.1 - 5.3
Novel HRV-derived metrics						
PIP (%)	72.0	± 6.3	75.0	± 6.7	66.3	± 7.2
α1	1.26	1.15 - 1.39	1.3	1.1 - 1.4	1.3	1.1 - 1.4

Panel A

	Unadjusted - 24-hour			Unadjusted - Daytime			Unadjusted - Nighttime		
	HR	95% CI	P-value	HR	95% CI	P-value	HR	95% CI	P-value
Time-domain HRV analysis									
RMSSD	**1.02**	**(1.00 - 1.03)**	**<0.01**	**1.02**	**(1.01 - 1.03)**	**<0.001**	1.01	(1.00 - 1.02)	0.05
pNN50	**1.03**	**(1.01 - 1.05)**	**<0.01**	**1.03**	**(1.01 - 1.05)**	**<0.01**	1.01	(0.99 - 1.03)	0.31
Frequence-domain HRV analysis									
LF / HF	**0.92**	**(0.86 - 0.99)**	**0.02**	**0.92**	**(0.84 - 0.99)**	**0.03**	0.94	(0.87 - 1.01)	0.06
Novel HRV-derived metrics									
PIP	**1.04**	**(1.01 - 1.07)**	**<0.01**	**1.04**	**(1.01 - 1.07)**	**<0.01**	**1.03**	**(1.01 - 1.06)**	**<0.01**
α1	**0.18**	**(0.07 - 0.45)**	**<0.01**	**0.17**	**(0.08 - 0.40)**	**<0.01**	0.49	(0.21 - 1.14)	0.10

Panel B

Online Figure 3. Additional HRV parameter results based on three distinct assessment timelines.

A. Baseline heart rate variability (HRV) parameters derived from three different time periods: 24-hour, daytime, and nighttime.
B. Univariate Cox proportional hazard models for incident AF over an 18-year follow-up, based on the timeline of the HRV parameters.

	Variable of Interest			Potential Moderator (Male Sex)			Interaction analysis		
	HR	95% CI	P-value	HR	95% CI	P-value	HR	95% CI	P-value
24-hour EKG Holter Parameters									
**Decreased heart rate**	1.76	(1.06 - 2.93)	0.03	2.06	(1.20 - 3.53)	<0.01	0.65	(0.32 - 1.34)	**0.25**
**Increased PAC burden**	2.83	(1.47 - 5.45)	<0.01	1.89	(1.28 - 2.78)	<0.01	0.52	(0.20 - 1.36)	**0.18**
**Increased PNN50**	1.94	(1.16 - 3.23)	0.01	1.92	(1.21 - 3.05)	<0.01	0.75	(0.36 - 1.54)	**0.42**
**Decreased α1**	2.45	(1.47 - 4.08)	<0.001	1.93	(1.21 - 3.07)	<0.01	1.14	(0.54 - 2.38)	**0.73**
**Increased PIP**	2.19	(1.14 - 4.22)	0.02	1.70	(0.75 - 3.84)	0.21	1.04	(0.42 - 2.57)	**0.94**

Online Figure 4. Interaction analysis between independent predictors of AF occurrence and male sex.

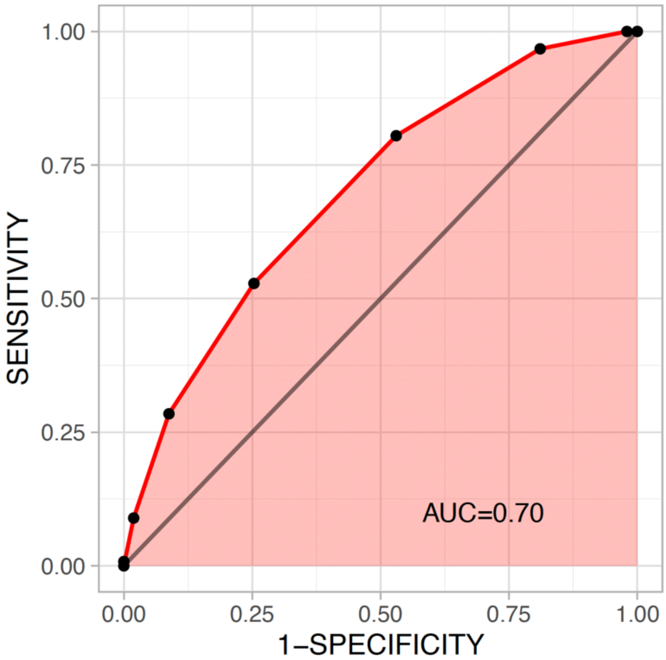

Online Figure 5. Receiver operating characteristic curve of the PROOF-AF risk score for prediction of AF occurrence during a long-term follow-up in general population.

AUC: area under the curve.

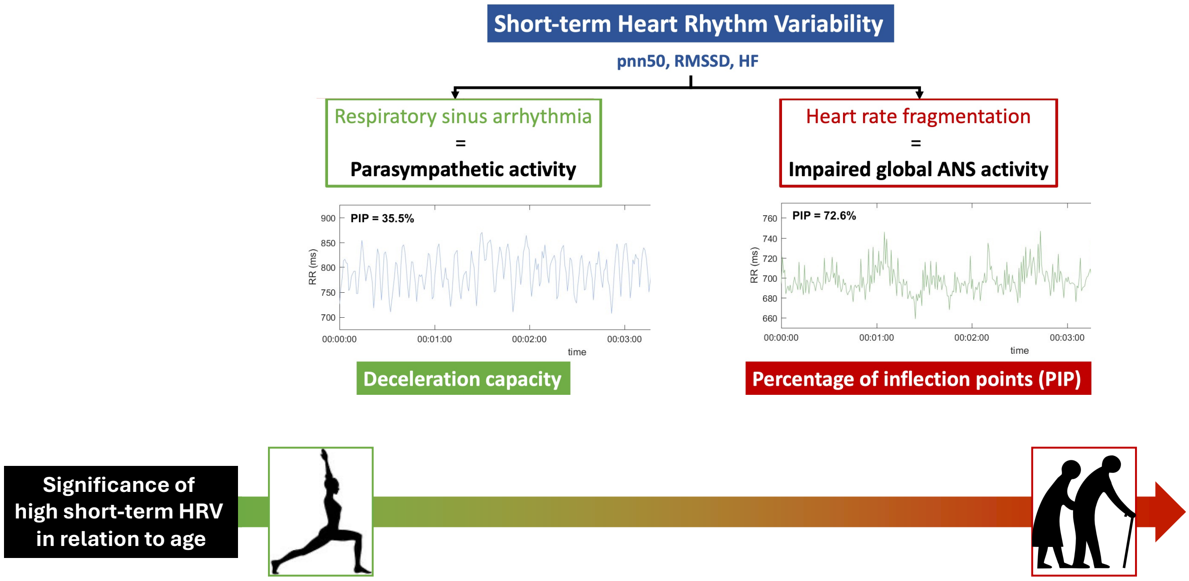

Online Figure 6. Graphical abstract of the pathophysiological hypothesis supported by the results of the PROOF-AF study. An increase in short-term heart rate variability is due to vagal activity in the young and active population, whereas it results from heart rhythm fragmentation and breakdown of autonomic nervous system function in the elderly population.
